# A Comparative Assessment of Cooling Center Preparedness across Twenty-Five U.S. Cities

**DOI:** 10.3390/ijerph18094801

**Published:** 2021-04-30

**Authors:** Kyusik Kim, Jihoon Jung, Claire Schollaert, June T. Spector

**Affiliations:** 1 Department of Geography, Florida State University, Tallahassee, FL 32306, USA; kkim17@fsu.edu; 2 Department of Environmental and Occupational Health Sciences, University of Washington, Seattle, WA 98195, USA; cscholla@uw.edu (C.S.); spectj@uw.edu (J.T.S.)

**Keywords:** cooling center, preparedness, heat waves, extreme heat, population coverage, subpopulation groups, disparity

## Abstract

Cooling centers have played a significant role in reducing the risks of adverse health impacts of extreme heat exposure. However, there have been no comparative studies investigating cooling center preparedness in terms of population coverage, location efficiency, and population coverage disparities among different subpopulation groups. Using a catchment area method with a 0.8 km walking distance, we compared three aspects of cooling center preparedness across twenty-five cities in the U.S. We first calculated the percentage of the population covered by a single cooling center for each city. Then, the extracted values were separately compared to the city’s heat indexes, latitudes, and spatial patterns of cooling centers. Finally, we investigated population coverage disparities among multiple demographics (age, race/ethnicity) and socioeconomic (insurance, poverty) subpopulation groups by comparing the percentage of population coverage between selected subpopulation groups and reference subpopulation groups. Our results showed that cooler cities, higher latitude cities, and cities with dispersed cooling centers tend to be more prepared than warmer cities, lower latitude cities, and cities with clustered cooling centers across the U.S. Moreover, older people (≥65) had 9% lower population coverage than younger people (≤64). Our results suggest that the placement of future cooling centers should consider both the location of other nearby cooling centers and the spatial distribution of subpopulations to maximize population coverage and reduce access disparities among several subpopulations.

## 1. Introduction

Multiple studies have projected more intense, more frequent, and longer-lasting heat waves in the future [1,2,3]. Enhanced and elongated heat waves will likely increase future mortality and morbidity from cardiovascular, respiratory, heat-related, and renal diseases [4,5,6]. However, most heat-related health outcomes are preventable. McGinnis and Foege [7] and Mokdad et al. [8] showed that more than 50% of all deaths could be prevented through behavior change alone.

Air conditioning may be the most effective way to prevent heat-related illnesses [9]. Bouchama et al.’s [10] meta-analysis indicates that individuals with home air conditioning units and those who visit other air-conditioned places have a 77% and 66% lower likelihood of heat-related mortality than their counterparts, respectively. Despite the protective effects of residential air conditioning, it is often unaffordable due to high implementation, maintenance, and running costs [11]. According to the U.S. Census Bureau’s American Housing Survey (AHS) conducted in 2019, approximately 28.6% of housing units in the U.S. did not have any type of air conditioning. Several subpopulations, who are already disproportionately affected by heat waves due to limited financial and social resources, are more likely to lack air conditioning units [12,13,14]. For instance, the same survey showed that the percentage of Black and Hispanic households with central air conditioning were lower than those of White and non-Hispanic households (White: 72.2%, Black: 70.6%, non-Hispanic: 72.4%, Hispanic: 65.1%). Some are also unable to use low-cost cooling devices such as electric fans due to energy costs [12]. For this reason, previous studies have highlighted the importance of public cooling spaces over private residential air conditioning [12,15].

Many state and federal governments are preparing their adaptation plans (e.g., CDC’s building resilience against climate effect program) to mitigate the impact of heat waves based on a heat vulnerability index and early warning systems. Cooling centers would be the integral part of the heat adaptation strategy and warning system [16]. Generally, cooling centers are implemented and operated by a variety of stakeholders including municipalities, fire departments, county agencies, and non-profit organizations, using existing facilities or buildings such as community centers, senior centers, and libraries [17]. Although these facilities help to reduce negative impacts on human health, the locations of cooling centers are not always optimized to maximize access [9]. Furthermore, the locations do not consider the spatial distribution of different demographic and socioeconomic subpopulation groups. Given that each subpopulation group has a different level of adaptive capacity, it would be beneficial to take into account different subpopulation groups when developing a city-specific strategic allocation of cooling centers [15].

Several studies have focused on the optimal location of cooling centers [9,15]. To measure the accessibility or population coverage of cooling centers, two methods have been commonly used: a catchment area method [18] and a shortest path approach [17]. The former approach measures the level of access by counting the number of people within a given catchment area defined by a certain distance or time. This approach assumes that anyone within a catchment area can have an equal level of access to a facility. In other words, there is no differentiation of proximity within the catchment area [19]. The latter approach estimates the level of accessibility using a given time or distance from the demand location (e.g., cooling center users) to the nearest facility (e.g., cooling centers), assuming that access to one facility is sufficient [20]. Since we consider the number of people who can walk to a cooling center within a given time or distance, the catchment area method is a better measure to evaluate cooling centers’ population coverage level than the shortest path approach.

Some studies have also investigated disparities in access to cooling centers faced by multiple subpopulation groups, rooted in differences in adaptive capacity among groups [17,21,22]. Voelkel et al. [22] reported that Black people have better access to public cooling centers, whereas older adults ≥ 65 years have less access in Portland, OR. Nayak et al. [17] also found that more vulnerable census tracts have better accessibility compared to less vulnerable census tracts using a heat vulnerability index covering multiple demographic (age, nativity, race, ethnicity) and socioeconomic variables (employment, language, income, housing, and environmental characteristics).

Regional characteristics such as location (i.e., latitude), heat index, and spatial patterns of cooling centers may partially influence the level of cooling center preparedness. Previous studies suggested that the cities at high latitudes are less prepared for extreme heat than the cities at low latitudes in terms of physiological and technological adaptations [23,24,25]. Multiple studies documented high mortality and morbidity rates in high latitude cities as a result of heat exposure as well [24,25,26]. In addition, some studies indicated that a dispersed distribution of facilities may provide more efficient population coverage than a clustered distribution [27,28].

To the best of our knowledge, there is no comparative study examining inter-city cooling center preparedness. The present study compares three aspects of cooling center preparedness: population coverage, location efficiency, and population coverage disparities among multiple subpopulations in twenty-five U.S. cities. The study has three main objectives. First, we compare population coverage levels across twenty-five cities by calculating the percentage of the city population covered by a single cooling center of each city. Second, we investigate the relationship between population coverage level and regional characteristics (i.e., latitude, HI, spatial patterns of cooling centers). Third, we examine disparities in the level of population coverage among five demographic and socioeconomic subpopulation groups.

## 2. Data and Methodology

**Figure 1:**
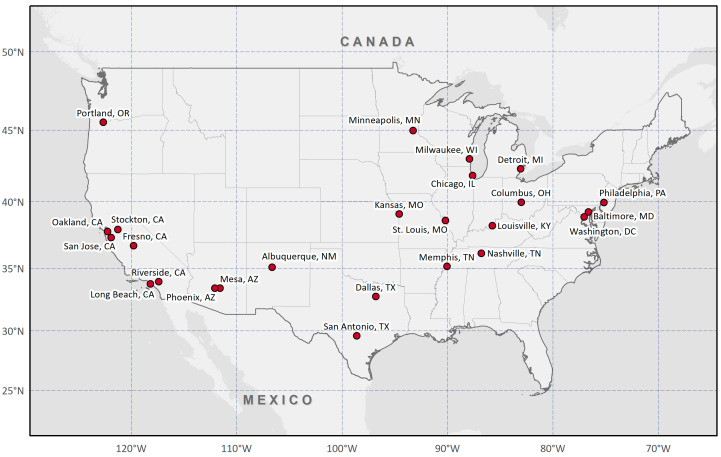
Twenty-five cities included in the analysis based on official data availability and population (>300,000).

This study analyzed data from twenty-five cities that have more than 300,000 people and provide official cooling center locations through their government websites or geographic information system portals in the U.S. (Figure 1): Albuquerque, NM; Baltimore, MD; Chicago, IL; Columbus, OH; Dallas, TX; Detroit, MI; Fresno, CA; Kansas City, MO; Long Beach, CA; Louisville, KY; Memphis, TN; Mesa, AZ; Milwaukee, WI; Minneapolis, MN; Nashville, TN; Oakland, CA; Philadelphia, PA; Phoenix, AZ; Portland, OR; Riverside, CA; St. Louis, MO; San Antonio, TX; San Jose, CA; Stockton, CA; Washington, DC.

### 2.1. Data

#### 2.1.1. Cooling Center

Cooling centers are air-conditioned facilities or buildings, such as libraries, community centers, and senior centers, which are designed to provide people with food, drink, and medical services during hot summer days [16,17]. To maintain consistency across cities, we only included cooling centers listed as cooling shelters, libraries, community centers, and senior centers, and excluded other private facilities such as supermarkets and theaters. We collected the locations of cooling centers from two official data sources: (1) city government’s websites and (2) government geographic information system portals. More detailed information on data sources and the location of cooling centers can be found in Table S1 and Figure S1.

#### 2.1.2. Weather

Monthly level Parameter-elevation Regressions on Independent Slopes Model (PRISM) data were used to calculate the 30-year daily average maximum HI of study areas. PRISM data were developed to interpolate climate data in physiographically complex landscapes based on 12,937 stations for precipitation, 9783 for maximum temperature, and 9871 for minimum temperature [29]. The data provide spatially and temporally consistent 800 m resolution weather data for the continental U.S. from 1895 to the present, using climate-elevation regression and station weighting functions. We used each city’s centroid to extract daily maximum temperature and relative humidity from May through September for 30 years (1990 to 2019). We then calculated the daily average maximum HI using Equation (1) [30]. We converted the unit of HI from Fahrenheit to Celsius using Equation (2).

HI (°F) = −42.379 + 2.049015T + 10.143331R − 0.224755TR − 6.83783 × 10^−3^T^2^ − 5.481717 × 10^−2^R^2^ + 1.22874 × 10^−3^T^2^R + 8.5282 × 10^−4^TR^2^ − 1.99 × 10^−6^T^2^R^2^, (1)

°C = 5/9 × (°F − 32),(2)

where T is air temperature (°F) and R is relative humidity (%). F stands for Fahrenheit, and C stands for Celsius.

#### 2.1.3. Road Network

Road network data were obtained through the OSMnx package in Python [31]. The package provides various types of road networks by mode of transportation (e.g., car, bike, walk), extracted from the OpenStreetMap. Multiple studies have used this package to attain, analyze, and visualize complex transport-related variables (e.g., speed, travel time, distance) [32,33,34]. The present study downloaded administrative boundaries and walkable roads for all twenty-five cities. The downloaded street network data were then combined with the cooling center location data to calculate the areas within a given walking distance (i.e., 0.8 km).

#### 2.1.4. Demographic and Socioeconomic Subpopulations

The U.S. Census Bureau American Community Survey (ACS) 2015–2019 5-year estimates provide demographic and socioeconomic data at the census block group level. The main purpose of the ACS is to measure the annual change in a community’s demographic and socioeconomic characteristics of the U.S. population [35], supplying a wide range of demographic (e.g., age, sex, race/ethnicity) and socioeconomic variables (e.g., income, employment, education, housing). Based on previous research, we selected five of the most important demographic and socioeconomic characteristics associated with heat-related human health: age, race, ethnicity, income, and health insurance status [36,37,38,39,40,41,42,43]. Note that the list of the characteristics used in the analysis is not intended to be exhaustive, but rather representative of the main factors known to or suspected to be highly related to the impact of heat waves.

Some demographic factors are highly associated with an individual’s ability to adapt to extreme heat exposure. Previous studies have shown that people over 65 years are more vulnerable than other age groups due to their degraded ability to detect thirst and reduced autonomic ability to adjust skin blood flow [36,37,38]. Multiple studies have also demonstrated Blacks are at higher risk of mortality and morbidity as a result of heat exposure, which may be attributable to their relatively low socioeconomic status [39,40]. For example, the central AC prevalence among Black households was less than half of the prevalence among households of other races in Chicago, Detroit, Minneapolis, and Pittsburgh [43]. 

Socioeconomic status may also impact an individual’s vulnerability to extreme heat by increasing or decreasing adaptive capacity before/during/after extreme heat events. Multiple studies have shown that heat waves disproportionately affect low-income individuals [41] and those without health insurance [42]. With the consideration of these influential factors, we investigated five subpopulation groups: age (≥65 years), race (Black), ethnicity (Hispanic), poverty (below poverty level), and health insurance status (population uninsured).

### 2.2. Methodology

**Figure 2:**
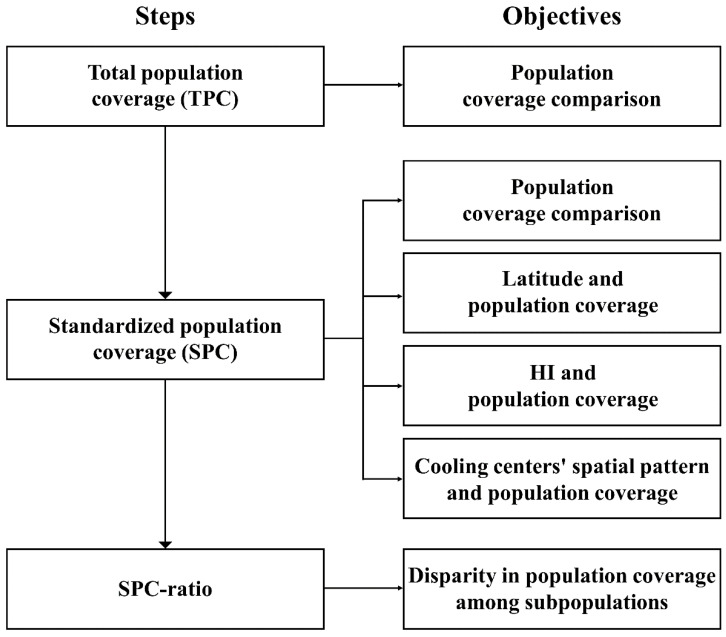
Schematic flow chart illustrating all procedures in this study.

Our analytic framework can be divided into three parts. First, we compare the level of cooling center population coverage across twenty-five U.S. cities. We then examine the relationship between population coverage and regional characteristics such as latitudes, HIs, and spatial pattern of cooling centers. Third, we explore access disparities in population coverage levels among various subpopulation groups. All procedures used in this study are briefly illustrated in Figure 2.

We employed a catchment area method to measure the level of cooling center population coverage for each city. This method counts the number of people within a given distance from a cooling center, assuming that anyone within the same catchment area has equal access to the cooling center. Based on existing literature [9,17,44], we selected a 0.8 km, which corresponds to a 15-min walking distance, catchment area. Since the boundary of the catchment area does not perfectly overlap with the boundaries of block groups, we estimated the population size based on the size of the overlapping area, assuming that the population is uniformly distributed throughout the block group [19]. For example, if a catchment area overlaps with 20% of the census block group with a population of 1000, we assumed that 200 people (1000 × 0.20) live in the overlapping area. To avoid double-counting people within the catchment areas derived from multiple cooling centers, we merged all separate catchment areas into one large catchment area (see Figure S2 for details).

To calculate the level of cooling center population coverage for each city, we devised two measures: total population coverage (TPC) and standardized population coverage (SPC). We calculated TPC by dividing the total population in catchment areas by the total population in the city and multiplying it by 100, which indicates the percentage of population covered by cooling centers within 0.8 km (Equation (3)). This measure is useful to directly compare the percentages of a total population covered by cooling centers between cities. However, this measure allows the cities with more cooling centers to always have a higher population coverage than the cities with fewer cooling centers. For example, Chicago, IL, with 99 cooling centers would have a better population coverage level than Fresno, CA, with 4 cooling centers. As an alternative, we used SPC which measures the percentage of population covered by a single cooling center by dividing the TPC by the number of cooling centers in the city (Equation (4)). This measure enabled us to compare the percentage of total population coverage per cooling center by city.

(3) $$\mathrm{TPC}=\frac{\mathrm{Total}\;\mathrm{number}\;\mathrm{of}\;\mathrm{people}\;\mathrm{in}\;\mathrm{catchment}\;\mathrm{areas}}{\mathrm{Total}\;\mathrm{number}\;\mathrm{of}\;\mathrm{people}\;\mathrm{in}\;a\;\mathrm{city}}\times 100,$$

(4) $$\mathrm{SPC}=\frac{\mathrm{TPC}}{\mathrm{Total}\;\mathrm{number}\;\mathrm{of}\;\mathrm{cooling}\;\mathrm{centers}\;\mathrm{in}\;a\;\mathrm{city}}.$$

We then examined the relationships between the SPCs and regional characteristics such as latitudes, HIs, and spatial patterns of cooling centers. Latitudes and HIs were examined with scatter plots and Pearson’s correlation coefficients, and the spatial pattern of cooling centers were investigated using the average nearest neighbor (ANN) ratio. The ANN ratio is commonly used to measure the degree of clustering or dispersion of events by comparing the actual distance and the hypothetical distance extracted from a random distribution [45]. The ratios near zero, near one, and greater than one are likely to indicate clustering, random distribution, and dispersion, respectively. We calculated an ANN ratio by dividing the observed average nearest distance between two cooling centers by the expected average nearest distance when they are randomly distributed. In this process, we generated referenced random distributions following population density, assuming that cooling centers are located in more populated areas.

Finally, we investigated disparities in population coverage levels among multiple subpopulation groups by comparing the SPCs between selected subpopulation groups and reference subpopulation groups. We selected a different reference subpopulation group for each selected subpopulation group: population ≥ 65 years (reference: population ≤ 64), Black (non-Black), Hispanic (non-Hispanic), population with insurance (population without insurance), and population below poverty level (population above poverty level). We respectively calculated SPCs for the selected subpopulation groups and their reference groups. Then, we divided the SPC of the selected subpopulation group by the SPC of the reference subpopulation group to find the SPC-ratio between the two groups (Equation (5)). If two groups have the same level of population coverage, the SPC-ratio is one. On the other hand, when the SPC of the selected subpopulation group is lower than the SPC of the reference group, the SPC-ratio is lower than 1. This means the selected subpopulation has a lower population coverage level compared to the reference group. Similarly, when the SPC-ratio is higher than 1, the population coverage level for the selected subpopulation group is higher than the one for the reference subpopulation group. Since these selected subpopulation groups are more likely to experience higher heat-related health impacts, we postulated that they should have a higher population coverage level compared to its counterpart (reference subpopulation).

(5) $$\mathrm{SPC}-\mathrm{ratio}=\frac{\mathrm{SPC}\;\mathrm{of}\;\mathrm{selected}\;\mathrm{subpopulation}}{\mathrm{SPC}\;\mathrm{of}\;\mathrm{reference}\;\mathrm{subpopulation}}.$$

## 3. Results

### 3.1. Descriptive Summary

Table 1 shows the descriptive summary of twenty-five study areas. Note that some block group boundaries do not fall within city limits, which could underestimate or overestimate city populations. Among the study areas, Chicago, IL, (2.7 million people) and Riverside, CA, (0.3 million people) were the most and least populated cities, respectively, with six cities over 1 million, fourteen cities between 0.5 and 1 million, and five cities less than 0.5 million. Approximately, 10% to 17% of the population were ≥65 years of age across all cities. Blacks were the majority race in Baltimore, MD (62.4%), Detroit, MI (78.3%), and Memphis, TN (64%), whereas San Jose, CA (3.0%), Mesa, AZ (4.0%), and Albuquerque, NM (3.2%), had the lowest proportion of Black populations. More than 50% of the population in Riverside, CA (54.2%), and San Antonio, TX (63.6%), identified as Hispanic, while less than 5% in St. Louis, MO (4.0%), identified as Hispanic. The percent of population below the federal poverty level ranged from 8.7% (San Jose, CA) to 35.0% (Detroit, MI), and the uninsured ranged from 3.7% (Washington, DC) to 23.2% (Dallas, TX). HIs ranged from 24.7 °C (Oakland, CA) to 36.1 °C (Phoenix, AZ), though Oakland (37° N) and Phoenix (33.5° N) are located in similar latitudes. More details on the spatial patterns of HI can be found in Figure S3. For cooling centers, Chicago, IL, and Washington, DC, have over fifty cooling centers while Fresno, CA, has only four.

### 3.2. Population Coverage Level Comparison between Cities

**Figure 3:**
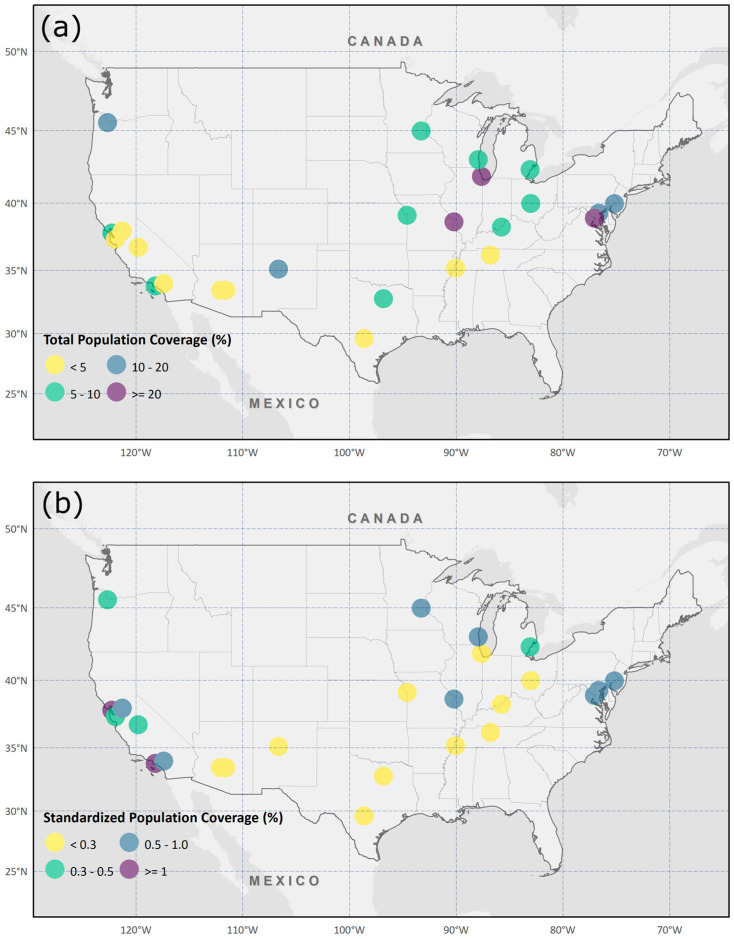
TPCs and SPCs of twenty-five study areas. (**a**) The spatial distribution of TPCs and (**b**) the spatial distribution of SPCs.

Figure 3a illustrates TPCs with colored circles. TPCs show the percentage of the total population within all catchment areas in a city. We calculated the percentage of people within the walking distance (i.e., 0.8 km) from cooling centers. Washington, DC (57.5%), and Fresno, CA (1.9%), had the highest and lowest coverage, respectively, with an average of 10.3% and a standard deviation of 11.9% over twenty-five cities. St. Louis, MO (27.1%), and Chicago, IL (25.9%), also had relatively high TPCs, while Phoenix, AZ (3.0%), Nashville, TN (2.7%), Mesa, AZ (2.1%), and San Jose, CA (2.1%), had low TPCs. The cities where the TPC is greater than 20% tended to be located in the midwestern and eastern U.S.

As mentioned in the methodology, assessing population coverage levels without considering the number of cooling centers could provide misleading information since the cities having more cooling centers would have higher TPCs. To standardize, we calculated SPC by dividing TPC by the number of cooling centers to represent the percentage of the total population covered by a single cooling center in a city. Figure 3b shows SPCs using colored circles. Oakland, CA (1.4%), and Long Beach, CA (1.4%), were ranked high and Phoenix, AZ (0.1%), San Antonio, TX (0.1%), and Dallas, TX (0.1%), were ranked low with an average of 0.5% and a standard deviation of 0.4% over twenty-five cities. Interestingly, southern states with high HI such as Arizona, Texas, and New Mexico had low SPCs while western coast, midwestern, and northeastern states with low HI such as California, Oregon, and New York had high SPCs. Readers can refer to Table S2 for detailed information on each city’s TPC and SPC.

### 3.3. The Association between Latitude/HI and SPC

**Figure 4:**
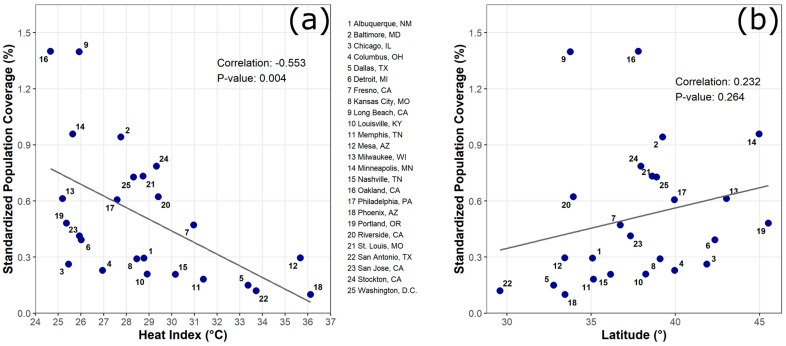
Associations of SPC with cities’ location characteristics. (**a**) Correlation between SPC and heat index and (**b**) correlation between SPC and latitude.

The associations between SPCs and both HI and latitude were explored using scatter plots and Pearson’s correlation coefficients (Figure 4). We found a negative association between HIs and SPCs (R = −0.553, *p*-value = 0.004) (Figure 4a). This suggests that the cities having higher HIs tend to have a lower level of population coverage (low SPCs). For example, the cities where HIs are greater than 33 °C (e.g., Dallas, TX; San Antonio, TX; Mesa, AZ; Phoenix, AZ) tended to have lower SPCs than the cities where HIs are lower than 26 °C (e.g., Oakland, CA; Long Beach, CA). While the association between SPC and latitude was not statistically significant, a positive relationship was found (R = 0.232, *p*-value = 0.264) (Figure 4b).

### 3.4. The Association between ANN Ratio and SPC

**Figure 5:**
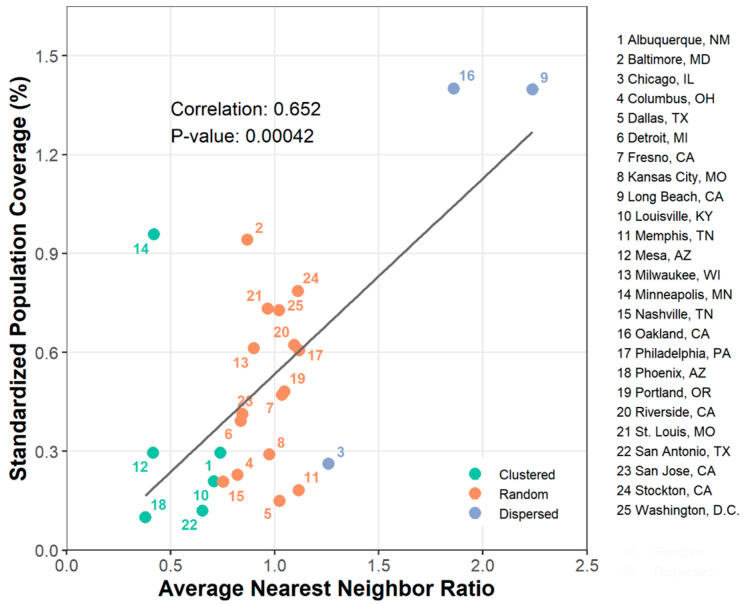
Association between SPC and ANN ratio. Green, orange, and blue indicate the cities where cooling centers were clustered, randomly distributed, and dispersed, respectively.

Figure 5 presents the association between ANN ratios and SPCs. As previously described, ratios near zero, near one, and greater than one are likely to indicate clustering, random distribution, and dispersion, respectively. We found a clustering pattern in six cities (Albuquerque, NM; Louisville, KY; Mesa, AZ; Minneapolis, MN; Phoenix, AZ; San Antonio, TX) and a dispersion pattern in three cities (Chicago, IL; Long Beach, CA; Oakland, CA). The Pearson’s correlation coefficient represented a positive relationship between ANN ratios and SPCs (R = 0.652, *p*-value < 0.001). This suggests that the cities where cooling centers are clustered may provide better population coverage levels compared to the cities where they are dispersed.

### 3.5. Disparity in Population Coverage Level for Subpopulation Groups

The disparity in population coverage levels for selected subpopulation groups was assessed by the SPC-ratio. SPC-ratio values greater and less than one represent higher and lower population coverage levels of selected subpopulations relative to reference subpopulations, respectively. An SPC-ratio value of one indicates the equal level of population coverage between two groups.

**Figure 6:**
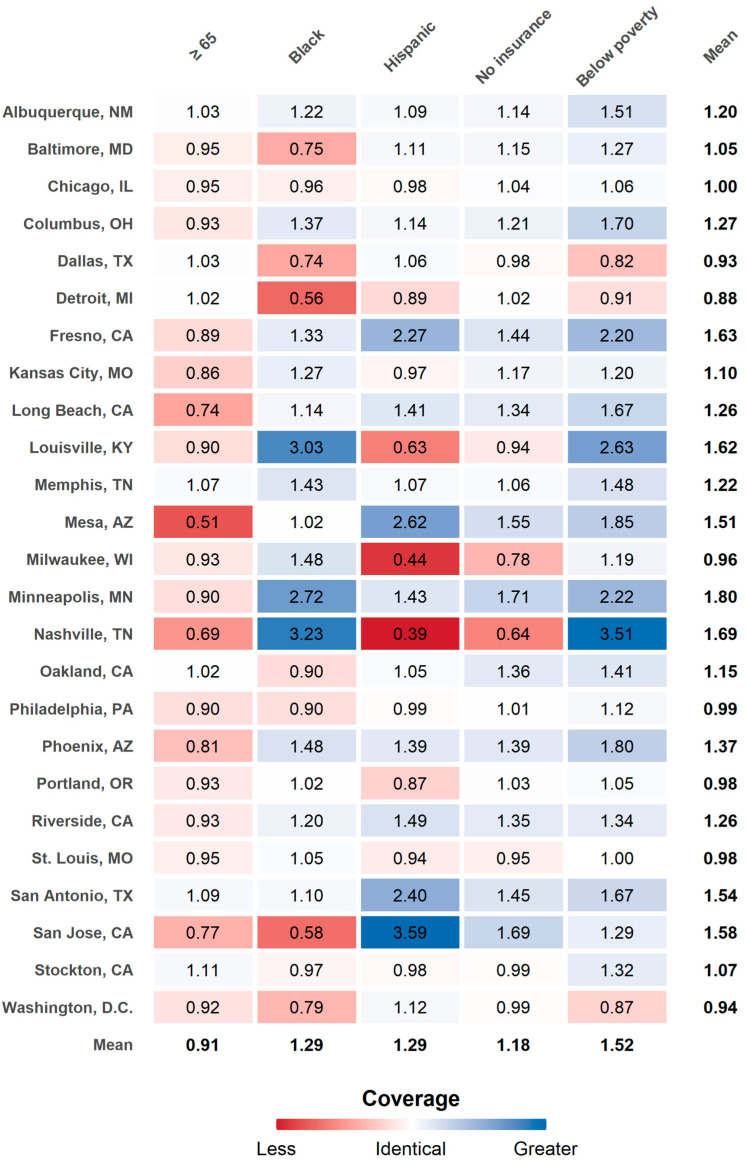
Subpopulation group comparisons with standardized population coverage ratios. Red and blue colors respectively represent less and greater service coverage for selected subpopulation groups compared to reference subpopulation groups.

Figure 6 illustrates which subpopulation groups and cities are more (low SPC-ratio; red color) or less (high SPC-ratio; blue color) vulnerable in terms of population coverage. Averaged across cities, the results showed that individuals ≥ 65 years have 0.91 times lower population coverage relative to individuals ≤ 64 years. In other words, older adults (≥65 years) were 9% less covered by cooling centers than younger adults (≤64 years). The SPC-ratio for Black and Hispanic subpopulation groups varied by cities. For example, while San Jose, CA showed low SPC-ratio for Black (0.58) and high SPC-ratio for Hispanic (3.59), Louisville, KY (Black: 3.03; Hispanic: 0.63), Milwaukee, WI (Black: 1.48; Hispanic: 0.44), and Nashville, TN (Black: 3.23; Hispanic: 0.39), showed an opposite pattern.

From the inter-city comparison, we observed Minneapolis, MN (1.80), and Detroit, MI (0.88), had the highest and lowest SPC-ratio with a mean of 1.24 across all cities. We found several subpopulations in some cities, including Detroit, MI (0.88), Dallas, TX (0.93), Washington, DC (0.94), Milwaukee, WI (0.96), Portland, OR (0.98), St. Louis, MO (0.98), and Philadelphia, PA (0.99), had a lower population coverage level than other cities on average. On the other hand, Albuquerque, NM (1.20), Memphis, TN (1.22), and San Antonio, TX (1.54), seemed to have a good level of population coverage for all subpopulations even though city-averaged SPC was not high. Some cities such as Nashville, TN (Black: 3.23; Hispanic: 0.39; Poverty: 3.51), and Louisville, KY (Black: 3.03; Hispanic: 0.63; Poverty: 2.63), had a substantially different level of population coverage to different subpopulation groups. Readers can find separate SPCs for selected subpopulation groups and reference groups in Table S3.

## 4. Discussion

This analysis found that the total population coverage per cooling center had a negative association with HI and a positive association with latitude. These results indicate that the cooler cities at high latitudes tend to be better prepared than the warmer cities at low latitudes. This contradicts our belief that the warmer cities at low latitudes are more prepared than the cooler cities at high latitudes for heat waves [24,46]. We suspect this may be related to a different regional rate of housing units with air conditioning. For example, the households in warmer cities at low latitudes are more likely to have home air conditioning compared to the households in cooler cities at high latitudes. According to the U.S. Census Bureau’s AHS, 92.2% of houses in Dallas, TX (2019), San Antonio, TX (2017), and Phoenix, AZ (2019), where the average daily HI is higher than 32 °C from May through September, have central air conditioning, whereas only 64.9% of houses in Detroit, MI, San Jose, CA, Long Beach, CA, Minneapolis, MN, Chicago, IL, Portland, OR, and Milwaukee, WI, where HI is lower than 26 °C, have central air conditioning. Since most of the houses in warmer cities at low latitudes have their own air conditioning at home, public cooling centers might not be needed in those cities.

This study also found disparities in population coverage by age. On average, the older subpopulations were approximately 9% less covered by cooling centers than the reference group (≤64) across all twenty-five cities. This result is well-aligned with Voelkel et al. [22], which showed older adults ≥ 65 tended to have less access to cooling centers in Portland, OR. Our result suggests that older people may struggle more to use cooling centers than other subpopulation groups due to their relatively limited population coverage level. Their limited mobility led by physical constraints may place another barrier to access to cooling centers [47,48]. Our result indicates that the spatial distribution of different subpopulations should be considered when determining future cooling center locations.

Moreover, this study found a strong positive association between spatial patterns of cooling centers and population coverage per cooling center. As White [27] noted, the dispersed distribution of facilities does not always ensure better population coverage than the clustered distribution. Nevertheless, our study showed that the cities with clustered cooling centers tended to have a lower population coverage level than the cities with dispersed cooling centers. This result might be caused by the overlapping catchment areas generated from two adjacent cooling centers. For example, if the distance between two cooling centers is less than 1.6 km, they should share their catchment areas based on the 0.8 km buffer used in this study. This could lead to redundancies in catchment areas and ultimately have smaller population coverage. On the other hand, if the distance between cooling centers is sufficiently distant (i.e., >1.6 km), each cooling center would not share its catchment area with other adjacent cooling centers and provide better population coverage. Our results suggest that it is necessary to allocate cooling centers in consideration of the distance between other adjacent cooling centers.

Finally, we exhibited substantially different inter-city and within-city subpopulation coverage levels. For example, some cities (e.g., Minneapolis, MN; Nashville, TN; Fresno, CA; Louisville, KY; San Jose, CA) seemed to have better subpopulation coverage than other cities (e.g., Detroit, MI; Dallas, TX; Washington, DC). We suspect this variation might be associated with the population density of cooling center locations. For example, if more cooling centers happen to be located near the densely populated areas of subpopulations, the city would have a better subpopulation coverage level. On the other hand, if more cooling centers happen to be placed near sparsely populated areas, this city would have a lower subpopulation coverage level. This large variation supports city-specific strategies for allocating cooling centers for extreme heat events.

Our findings align with Sustainable Development Goals (SDGs) proposed in the United Nation’s 2030 Agenda “Transforming Our World: The 2030 Agenda for Sustainable Development” [49]. The 17 SDGs aim to improve health and well-being, increase equity, and boost economic growth while tackling climate change. Our study helps ensure human health and well-being (Goal 3), increase equity (Goal 10), and prepare future climate change (Goal 13) by providing information that could inform equitable heat wave adaptation planning and healthcare resource allocation. Specifically, our results suggest that those cities with high HIs, at lower latitude, and with clustered cooling centers were relatively less well prepared than those cities with low HIs, at higher latitude, and with dispersed cooling centers. We also found that older people have a lower coverage level than other age groups. This study provides key information that can inform future cooling center planning approaches that consider population coverage and equity and align with SDGs.

There are at least three limitations in this study. First, multiple cooling centers likely located near city boundaries were not included in some cities (Baltimore, MD; Louisville, KY; Milwaukee, WI; Portland, OR; Riverside, CA) due to limited data availability. This could have possibly underestimated the population coverage level. However, since we considered 0.8 km as a catchment area of cooling centers, the potential underestimation would be very small. Second, while this analysis only considered 2019 and 2020 data, the location of cooling centers may change every year. However, we believe the overall result may not be significantly different from other years because most cooling centers use existing facilities such as public libraries, senior centers, or community centers which are fixed. Third, we calculated HIs with daily maximum temperature and minimum relative humidity. Because we did not use the corresponding humidity when maximum temperature was observed, there could be some differences between actual HIs and the calculated HIs. Despite these limitations, this study can help better understand cooling center preparedness of twenty-five cities in the U.S.

This study brings to light at least four future research questions. Our study showed that some cities (e.g., Long Beach, CA; Oakland, CA) had better population coverage levels than other cities (e.g., Dallas, TX; Phoenix, AZ). Future research could investigate common factors that make the difference between cities. Second, the large variation of multiple subpopulation groups within a city warrants further study. For example, our result showed that several cities (e.g., Nashville, TN; Louisville, KY; San Jose, CA) had extreme population coverage disparities for Black and Hispanic populations. Third, it would also be worthwhile to propose optimal locations of cooling centers while taking into account other private cooling center facilities such as supermarkets, shopping malls, or theaters. In addition, including empirical data sets such as health outcomes in the analysis could provide more robust results regarding the impact of cooling centers on the general public and subpopulation groups.

## 5. Conclusions

This paper assessed and compared cooling center preparedness across twenty-five U.S. cities. We showed that the level of population coverage per cooling center is negatively associated with HIs and positively related to latitudes. We also observed that the cities with dispersed cooling centers tended to have a better population coverage level than the cities with clustered cooling centers. Furthermore, this paper revealed that older people (≥65) had the least population coverage level across the research areas and subpopulations.

There are several implications of this research for public health practice and policy. First, establishing heat wave adaptation plans and strategies, which consider current locations of cooling centers and spatial distributions of vulnerable subpopulation groups, would help cover more vulnerable people and reduce disparities among subpopulation groups. Second, our results could help prioritize healthcare resource allocation planning for high-risk subpopulations, not only for cooling centers, but also other facilities such as emergency departments or hospitals. Third, our findings support the need for lower latitude and high HI cities to prepare and be ready for adverse health impacts from heat waves. Even though housing units in these cities are more likely to have air conditioning than other cities, this does not mean every housing unit has air conditioning. Some cooling center users live in more vulnerable places (e.g., places where people are afraid to take a walk or to open windows due to the fear of crime) and have less protection (e.g., insurance, air conditioning), and future extreme heat could disproportionately impact them. Therefore, we believe incorporation of information about cooling center locations and spatial distribution of populations at highest risk into adaptation and healthcare planning is critical for improving health equity and reducing the overall public health burden of the negative health effects of heat exposures.

## Figures and Tables

**Table 1:** Descriptive characteristics.

City	Heat Index (°C)	Cooling Center (Count)	Total Population	Age (%)		Black (%)		Hispanic (%)		Insurance (%)		Poverty Level (%)	
				≥65	≤64 (Ref)	Yes	No (Ref)	Yes	No (Ref)	No	Own (Ref)	Below	Above (Ref)
Albuquerque, NM	28.8	45	568,755	14.7	85.3	3.2	96.8	49.9	50.1	8.1	91.9	17.0	83.0
Baltimore, MD	27.8	11	609,032	13.6	86.4	62.4	37.6	5.3	94.7	6.6	93.4	21.2	78.8
Chicago, IL	25.5	99	2,712,529	12.4	87.6	29.5	70.5	28.8	71.2	9.7	90.3	18.4	81.6
Columbus, OH	27.0	29	913,582	10.5	89.5	28.1	71.9	6.1	93.9	8.8	91.2	19.0	81.0
Dallas, TX	33.4	45	1,357,894	10.5	89.5	23.9	76.1	41.4	58.6	23.2	76.8	18.6	81.4
Detroit, MI	26.0	24	674,841	13.6	86.4	78.3	21.7	7.7	92.3	8.4	91.6	35.0	65.0
Fresno, CA	31.0	4	549,961	11.7	88.3	7.1	92.9	49.3	50.7	8.3	91.7	25.0	75.0
Kansas City, MO	28.5	28	505,856	12.9	87.1	27.4	72.6	10.4	89.6	11.6	88.4	15.7	84.3
Long Beach, CA	25.9	5	469,937	11.5	88.5	12.6	87.4	42.6	57.4	8.5	91.5	16.7	83.3
Louisville, KY	28.9	25	658,837	15.1	84.9	23.1	76.9	5.5	94.5	5.3	94.7	15.3	84.7
Memphis, TN	31.4	18	677,513	12.6	87.4	64.0	36.0	7.0	93.0	13.7	86.3	24.5	75.5
Mesa, AZ	35.7	7	516,705	17.0	83.0	4.0	96.0	27.6	72.4	12.2	87.8	15.0	85.0
Milwaukee, WI	25.2	16	594,722	10.5	89.5	38.7	61.3	19.0	81.0	9.3	90.7	25.4	74.6
Minneapolis, MN	25.6	6	420,324	10.0	90.0	19.2	80.8	9.6	90.4	6.6	93.4	19.1	80.9
Nashville, TN	30.2	13	665,708	11.8	88.2	27.5	72.5	10.5	89.5	12.1	87.9	15.1	84.9
Oakland, CA	24.7	5	425,097	13.1	86.9	23.8	76.2	27.0	73.0	7.9	92.1	16.7	83.3
Philadelphia, PA	27.6	29	1,579,075	13.4	86.6	42.1	57.9	14.7	85.3	8.1	91.9	24.3	75.7
Phoenix, AZ	36.1	30	1,649,286	10.7	89.3	7.1	92.9	42.5	57.5	14.0	86.0	17.9	82.1
Portland, OR	25.4	27	655,855	12.9	87.1	5.8	94.2	9.9	90.1	6.5	93.5	13.7	86.3
Riverside, CA	29.4	7	329,396	10.7	89.3	6.1	93.9	54.2	45.8	9.5	90.5	13.9	86.1
St. Louis, MO	28.7	37	308,174	13.1	86.9	46.4	53.6	4.0	96.0	10.8	89.2	21.8	78.2
San Antonio, TX	33.7	25	1,589,745	11.9	88.1	7.0	93.0	63.6	36.4	16.3	83.7	17.3	82.7
San Jose, CA	25.9	5	1,060,954	12.5	87.5	3.0	97.0	31.5	68.5	5.1	94.9	8.7	91.3
Stockton, CA	29.3	5	329,698	12.5	87.5	10.6	89.4	43.8	56.2	6.9	93.1	18.0	82.0
Washington, DC	28.3	79	692,683	12.1	87.9	46.3	53.7	11.0	89.0	3.7	96.3	16.2	83.8
Average	28.8	25	820,646	12.3	87.7	25.9	74.1	24.9	75.1	9.6	90.4	18.8	81.2

## Data Availability

No new data were created or analyzed in this study.

## Supplementary Materials

The following are available online at https://www.mdpi.com/article/10.3390/ijerph18094801/s1, Figure S1: Cooling center locations and population densities, Table S1: Sources of cooling center in 25 cities, Figure S2: Illustration of catchment areas, Figure S3: Spatial distribution of heat index, Table S2: Total population coverage and standardized population coverage of study cities, Table S3: Separate SPCs for selected subpopulation groups and reference groups.

## References

[1] IPCC (2014). Climate Change 2014: Synthesis Report.

[2] Meehl G.A. (2004). More Intense, More Frequent, and Longer Lasting Heat Waves in the 21st Century. Science.

[3] Beniston M., Stephenson D.B., Christensen O.B., Ferro C.A.T., Frei C., Goyette S., Halsnaes K., Holt T., Jylhä K., Koffi B. (2007). Future Extreme Events in European Climate: An Exploration of Regional Climate Model Projections. Clim. Chang..

[4] Li M., Gu S., Bi P., Yang J., Liu Q. (2015). Heat Waves and Morbidity: Current Knowledge and Further Direction-a Comprehensive Literature Review. Int. J. Environ. Res. Public Health.

[5] Sherbakov T., Malig B., Guirguis K., Gershunov A., Basu R. (2018). Ambient Temperature and Added Heat Wave Effects on Hospitalizations in California from 1999 to 2009. Environ. Res..

[6] Cheng J., Xu Z., Bambrick H., Prescott V., Wang N., Zhang Y., Su H., Tong S., Hu W. (2019). Cardiorespiratory Effects of Heatwaves: A Systematic Review and Meta-Analysis of Global Epidemiological Evidence. Environ. Res..

[7] McGinnis J.M., Foege W.H. (1993). Actual Causes of Death in the United States. JAMA.

[8] Mokdad A.H. (2004). Actual Causes of Death in the United States, 2000. JAMA.

[9] Fraser A.M., Chester M.V., Eisenman D. (2018). Strategic Locating of Refuges for Extreme Heat Events (or Heat Waves). Urban Clim..

[10] Bouchama A., Dehbi M., Mohamed G., Matthies F., Shoukri M., Menne B. (2007). Prognostic Factors in Heat Wave-Related Deaths: A Meta-Analysis. Arch. Intern. Med..

[11] World Health Organization WHO Housing and Health Guidelines. https://www.who.int/publications-detail-redirect/9789241550376.

[12] Farbotko C., Waitt G. (2011). Residential Air-Conditioning and Climate Change: Voices of the Vulnerable. Health Promot. J. Aust..

[13] Hayden M.H., Brenkert-Smith H., Wilhelmi O.V. (2011). Differential Adaptive Capacity to Extreme Heat: A Phoenix, Arizona, Case Study. Weather Clim. Soc..

[14] Sampson N.R., Gronlund C.J., Buxton M.A., Catalano L., White-Newsome J.L., Conlon K.C., O’Neill M.S., McCormick S., Parker E.A. (2013). Staying Cool in a Changing Climate: Reaching Vulnerable Populations during Heat Events. Glob. Environ. Chang..

[15] Bradford K., Abrahams L., Hegglin M., Klima K. (2015). A Heat Vulnerability Index and Adaptation Solutions for Pittsburgh, Pennsylvania. Environ. Sci. Technol..

[16] Widerynski S., Schramm P.J., Conlon K.C., Noe R.S., Grossman E., Hawkins M., Nayak S.U., Roach M., Hilts A.S. (2017). Use of Cooling Centers to Prevent Heat-Related Illness: Summary of Evidence and Strategies for Implementation.

[17] Nayak S.G., Shrestha S., Sheridan S.C., Hsu W.-H., Muscatiello N.A., Pantea C.I., Ross Z., Kinney P.L., Zdeb M., Hwang S.-A.A. (2019). Accessibility of Cooling Centers to Heat-Vulnerable Populations in New York State. J. Transp. Health.

[18] Fraser A.M., Chester M.V., Eisenman D., Hondula D.M., Pincetl S.S., English P., Bondank E. (2017). Household Accessibility to Heat Refuges: Residential Air Conditioning, Public Cooled Space, and Walkability. Environ. Plan. B Urban Anal. City Sci..

[19] McGrail M.R., Humphreys J.S. (2009). Measuring Spatial Accessibility to Primary Care in Rural Areas: Improving the Effectiveness of the Two-Step Floating Catchment Area Method. Appl. Geogr..

[20] Horner M.W., Duncan M.D., Wood B.S., Valdez-Torres Y., Stansbury C. (2015). Do Aging Populations Have Differential Accessibility to Activities? Analyzing the Spatial Structure of Social, Professional, and Business Opportunities. Travel Behav. Soc..

[21] Berisha V., Hondula D., Roach M., White J.R., McKinney B., Bentz D., Mohamed A., Uebelherr J., Goodin K. (2017). Assessing Adaptation Strategies for Extreme Heat: A Public Health Evaluation of Cooling Centers in Maricopa County, Arizona. Weather Clim. Soc..

[22] Voelkel J., Hellman D., Sakuma R., Shandas V. (2018). Assessing Vulnerability to Urban Heat: A Study of Disproportionate Heat Exposure and Access to Refuge by Socio-Demographic Status in Portland, Oregon. Int. J. Environ. Res. Public Health.

[23] Basu R., Samet J.M. (2002). Relation between Elevated Ambient Temperature and Mortality: A Review of the Epidemiologic Evidence. Epidemiol. Rev..

[24] Kravchenko J., Abernethy A.P., Fawzy M., Lyerly H.K. (2013). Minimization of Heatwave Morbidity and Mortality. Am. J. Prev. Med..

[25] McGeehin M.A., Mirabelli M. (2001). The Potential Impacts of Climate Variability and Change on Temperature-Related Morbidity and Mortality in the United States. Environ. Health Perspect..

[26] Gao J., Sun Y., Liu Q., Zhou M., Lu Y., Li L. (2015). Impact of Extreme High Temperature on Mortality and Regional Level Definition of Heat Wave: A Multi-City Study in China. Sci. Total Environ..

[27] White A.N. (1979). Accessibility and Public Facility Location. Econ. Geogr..

[28] Ye H., Kim H. (2016). Locating Healthcare Facilities Using a Network-Based Covering Location Problem. GeoJournal.

[29] Daly C., Halbleib M., Smith J.I., Gibson W.P., Doggett M.K., Taylor G.H., Curtis J., Pasteris P.P. (2008). Physiographically Sensitive Mapping of Climatological Temperature and Precipitation across the Conterminous United States. Int. J. Climatol. J. R. Meteorol. Soc..

[30] Steadman R.G. (1979). The Assessment of Sultriness. Part I: A Temperature-Humidity Index Based on Human Physiology and Clothing Science. J. Appl. Meteorol. Climatol..

[31] Boeing G. (2017). OSMnx: New Methods for Acquiring, Constructing, Analyzing, and Visualizing Complex Street Networks. Comput. Environ. Urban Syst..

[32] Neukart F., Compostella G., Seidel C., von Dollen D., Yarkoni S., Parney B. (2017). Traffic Flow Optimization Using a Quantum Annealer. Front. ICT.

[33] Hofer C., Jäger G., Füllsack M. (2018). Large Scale Simulation of CO2 Emissions Caused by Urban Car Traffic: An Agent-Based Network Approach. J. Clean. Prod..

[34] Ren Y., Cheng T., Zhang Y. (2019). Deep Spatio-Temporal Residual Neural Networks for Road-Network-Based Data Modeling. Int. J. Geogr. Inf. Sci..

[35] U.S. Census Bureau (2020). Understanding and Using American Community Survey Data: What All Data Users Need to Know.

[36] Hall D.M., Xu L., Drake V.J., Oberley L.W., Oberley T.D., Moseley P.L., Kregel K.C. (2000). Aging Reduces Adaptive Capacity and Stress Protein Expression in the Liver after Heat Stress. J. Appl. Physiol..

[37] Hanna E., Tait P. (2015). Limitations to Thermoregulation and Acclimatization Challenge Human Adaptation to Global Warming. Int. J. Environ. Res. Public Health.

[38] Horowitz M., Robinson S.D.M., Sharma H.S. (2007). Heat shock proteins and the heat shock response during hyperthermia and its modulation by altered physiological conditions. Progress in Brain Research.

[39] Hansen A., Bi L., Saniotis A., Nitschke M. (2013). Vulnerability to Extreme Heat and Climate Change: Is Ethnicity a Factor?. Glob. Health Action.

[40] Madrigano J., Ito K., Johnson S., Kinney P.L., Matte T. (2015). A Case-Only Study of Vulnerability to Heat Wave–RelatedMortality in New York City (2000–2011). Environ. Health Perspect..

[41] O’Neill M.S. (2003). Modifiers of the Temperature and Mortality Association in Seven US Cities. Am. J. Epidemiol..

[42] Zhang Y., Nitschke M., Bi P. (2013). Risk Factors for Direct Heat-Related Hospitalization during the 2009 Adelaide Heatwave: A Case Crossover Study. Sci. Total Environ..

[43] O’Neill M.S. (2005). Disparities by Race in Heat-Related Mortality in Four US Cities: The Role of Air Conditioning Prevalence. J. Urban Health Bull. N. Y. Acad. Med..

[44] Kisner C., Mulder K., VanGessel B. (2012). Assessing Heat Vulnerability and Access to Cooling Centers in Detroit, Michigan.

[45] Clark P.J., Evans F.C. (1954). Distance to Nearest Neighbor as a Measure of Spatial Relationships in Populations. Ecology.

[46] Curriero F.C., Heiner K.S., Samet J.M., Zeger S.L., Strug L., Patz J.A. (2002). Temperature and Mortality in 11 Cities of the Eastern United States. Am. J. Epidemiol..

[47] DeVita P., Hortobagyi T. (2000). Age Causes a Redistribution of Joint Torques and Powers during Gait. J. Appl. Physiol..

[48] Song S., Geyer H. (2018). Predictive Neuromechanical Simulations Indicate Why Walking Performance Declines with Ageing: Computer Simulations of Elderly Gait. J. Physiol..

[49] United Nations (UN) (2015). Transforming Our World: The 2030 Agenda for Sustainable Development.

