# Supplementary material for: A Comparative Assessment of Cooling Center Preparedness across Twenty-Five U.S. Cities
Source: Int J Environ Res Public Health. 2021 Apr 30;18(9):4801. doi: 10.3390/ijerph18094801 (PMC8125005; doi:10.3390/ijerph18094801)
Supplement: Supplementary file 1 [file ijerph-18-04801-s001.zip › ijerph-1146694-supplementary.pdf]

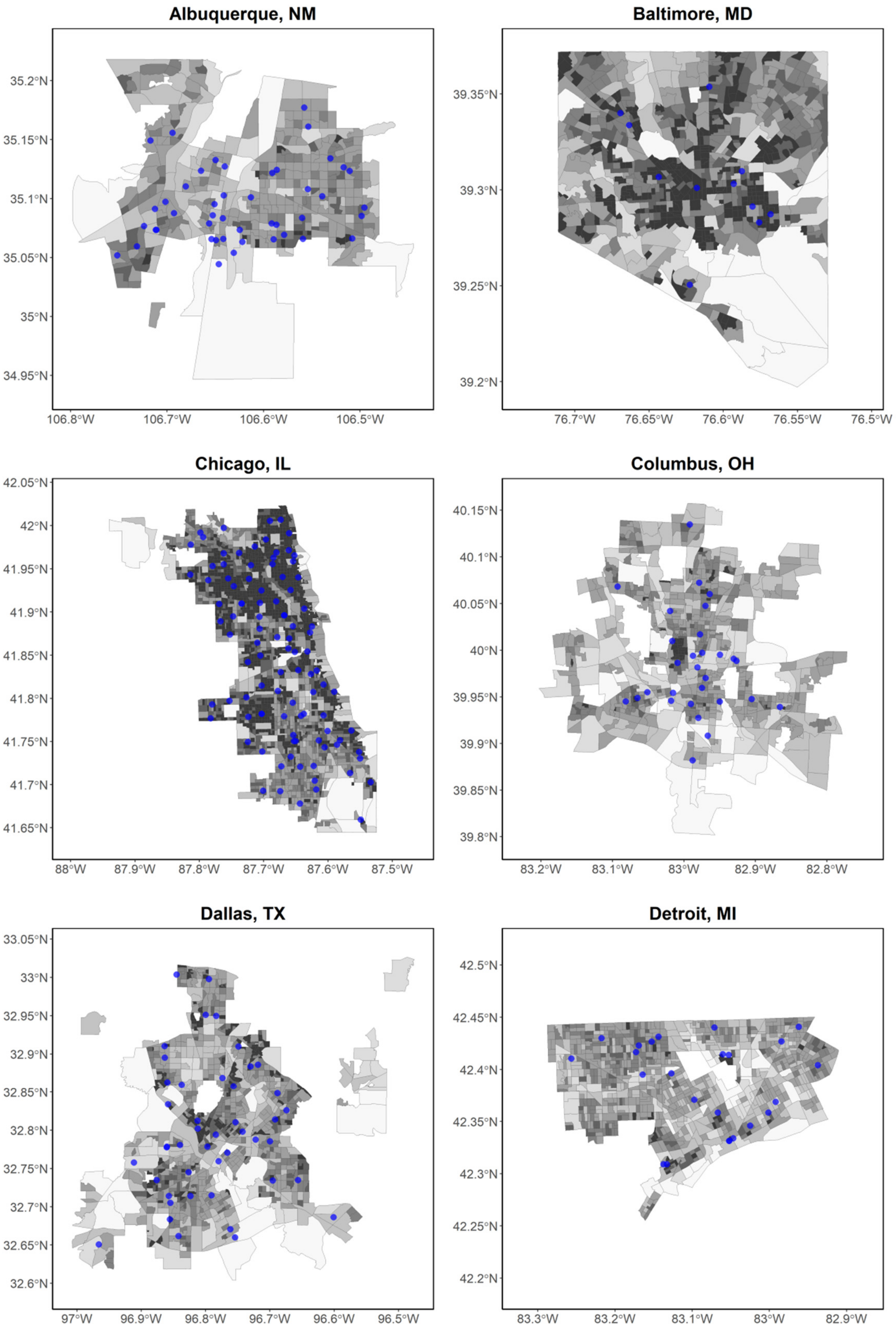

Albuquerque, NM
35.2°N
35.15°N
35.1°N
35.05°N
35°N
34.95°N
106.8°W
106.7°W
106.6°W
106.5°W
Baltimore, MD
39.35°N
39.3°N
39.25°N
39.2°N
76.7°W
76.65°W
76.6°W
76.55°W
76.5°W
Chicago, IL
42.05°N
42°N
41.95°N
41.9°N
41.85°N
41.8°N
41.75°N
41.7°N
41.65°N
88°W
87.9°W
87.8°W
87.7°W
87.6°W
87.5°W
Columbus, OH
40.15°N
40.1°N
40.05°N
40°N
39.95°N
39.9°N
39.85°N
39.8°N
83.2°W
83.1°W
83°W
82.9°W
82.8°W
Dallas, TX
33.05°N
33°N
32.95°N
32.9°N
32.85°N
32.8°N
32.75°N
32.7°N
32.65°N
32.6°N
97°W
96.9°W
96.8°W
96.7°W
96.6°W
96.5°W
Detroit, MI
42.5°N
42.45°N
42.4°N
42.35°N
42.3°N
42.25°N
42.2°N
83.3°W
83.2°W
83.1°W
83°W
82.9°W

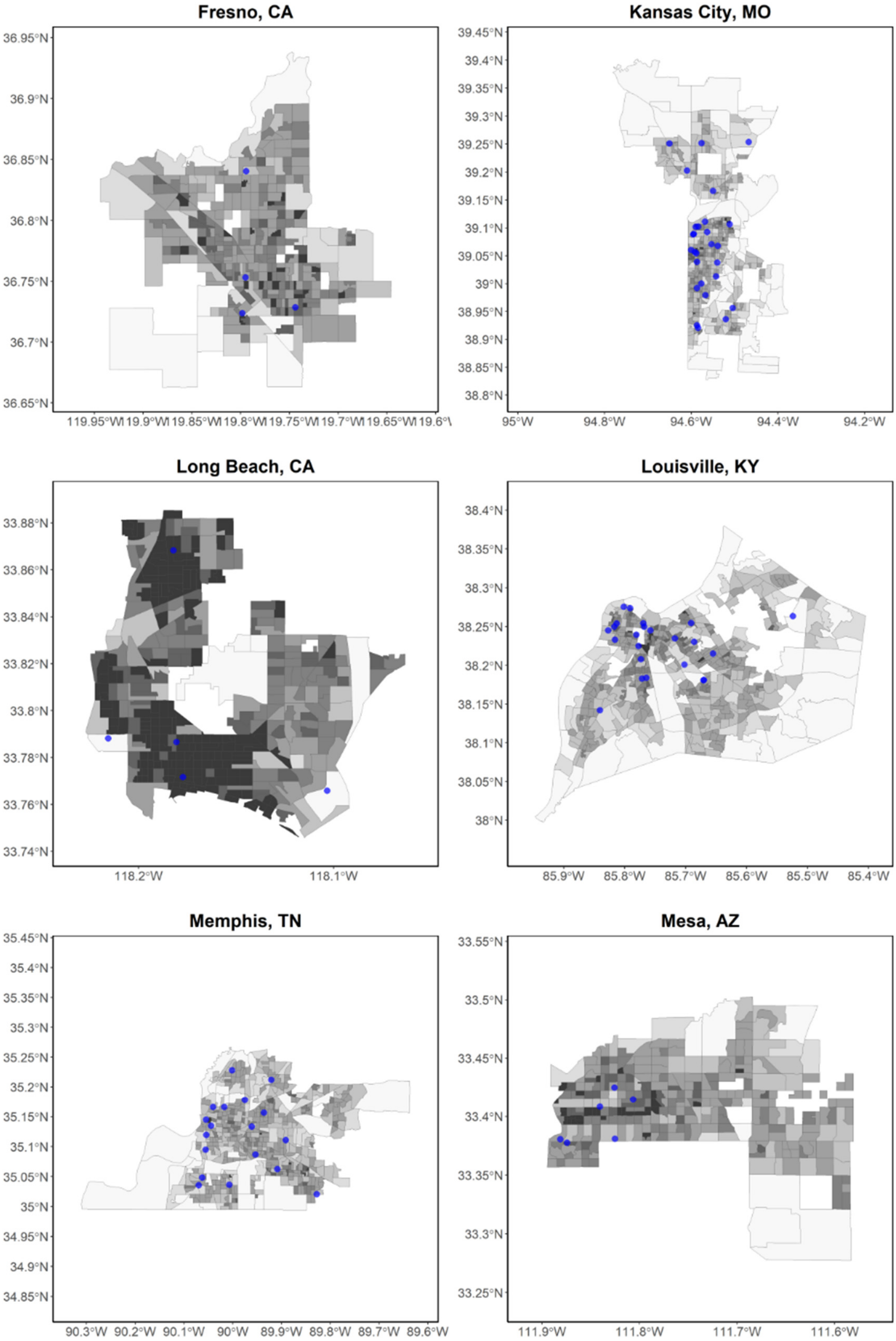

Fresno, CA
36.95°N
36.9°N
36.85°N
36.8°N
36.75°N
36.7°N
36.65°N
Kansas City, MO
39.45°N
39.4°N
39.35°N
39.3°N
39.25°N
39.2°N
39.15°N
39.1°N
39.05°N
39°N
38.95°N
38.9°N
38.85°N
38.8°N
95°W
94.8°W
94.6°W
94.4°W
94.2°W
Long Beach, CA
33.88°N
33.86°N
33.84°N
33.82°N
33.8°N
33.78°N
33.76°N
33.74°N
118.2°W
118.1°W
Louisville, KY
38.4°N
38.35°N
38.3°N
38.25°N
38.2°N
38.15°N
38.1°N
38.05°N
38°N
85.9°W
85.8°W
85.7°W
85.6°W
85.5°W
85.4°W
Memphis, TN
35.45°N
35.4°N
35.35°N
35.3°N
35.25°N
35.2°N
35.15°N
35.1°N
35.05°N
35°N
34.95°N
34.9°N
34.85°N
90.3°W
90.2°W
90.1°W
90°W
89.9°W
89.8°W
89.7°W
89.6°W
Mesa, AZ
33.55°N
33.5°N
33.45°N
33.4°N
33.35°N
33.3°N
33.25°N
111.9°W
111.8°W
111.7°W
111.6°W

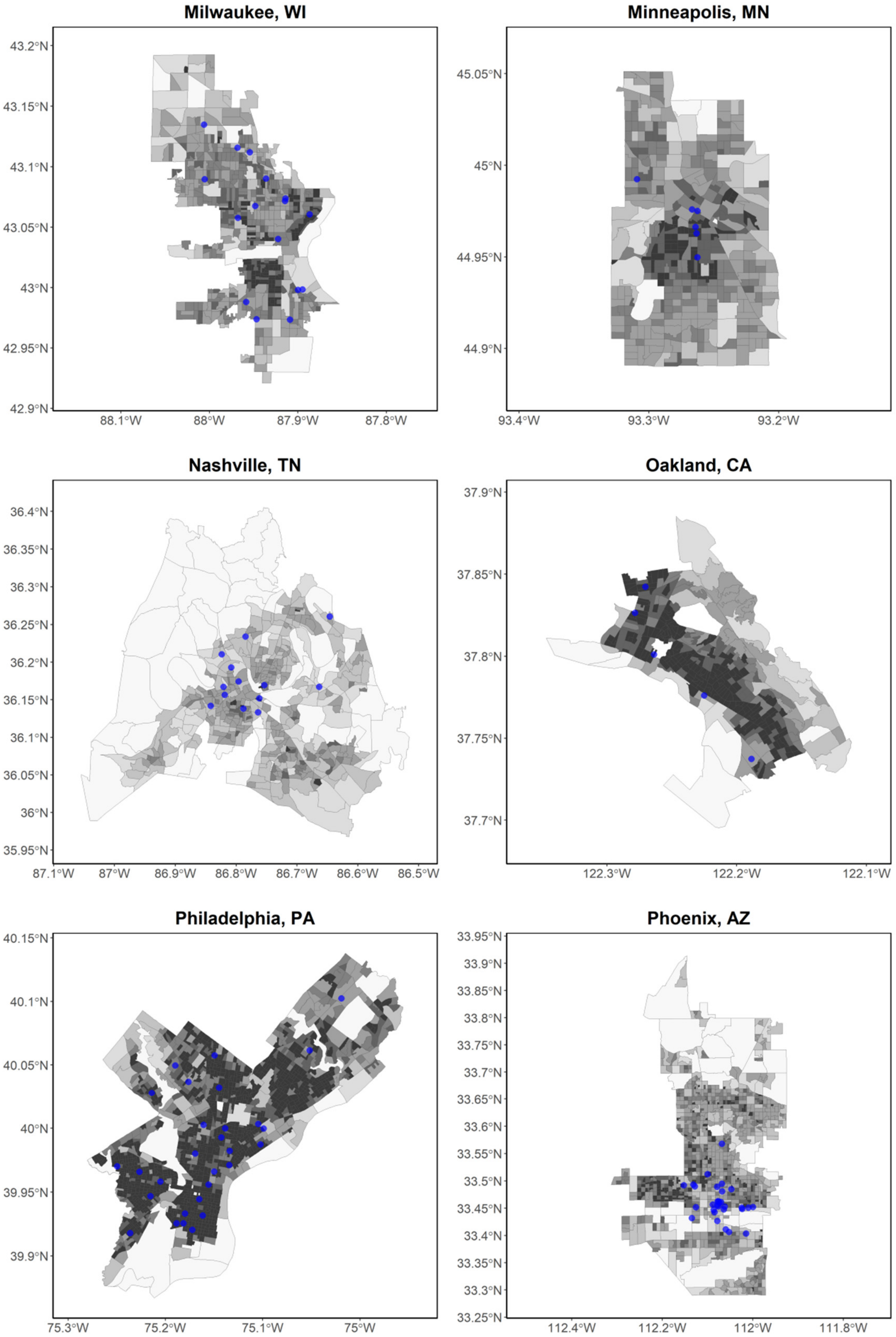
Milwaukee, WI
43.2°N
43.15°N
43.1°N
43.05°N
43°N
42.95°N
42.9°N
88.1°W
88°W
87.9°W
87.8°W
Minneapolis, MN
45.05°N
45°N
44.95°N
44.9°N
93.4°W
93.3°W
93.2°W
Nashville, TN
36.4°N
36.35°N
36.3°N
36.25°N
36.2°N
36.15°N
36.1°N
36.05°N
36°N
35.95°N
87.1°W
87°W
86.9°W
86.8°W
86.7°W
86.6°W
86.5°W
Oakland, CA
37.9°N
37.85°N
37.8°N
37.75°N
37.7°N
122.3°W
122.2°W
122.1°W
Philadelphia, PA
40.15°N
40.1°N
40.05°N
40°N
39.95°N
39.9°N
75.3°W
75.2°W
75.1°W
75°W
Phoenix, AZ
33.95°N
33.9°N
33.85°N
33.8°N
33.75°N
33.7°N
33.65°N
33.6°N
33.55°N
33.5°N
33.45°N
33.4°N
33.35°N
33.3°N
33.25°N
112.4°W
112.2°W
112°W
111.8°W

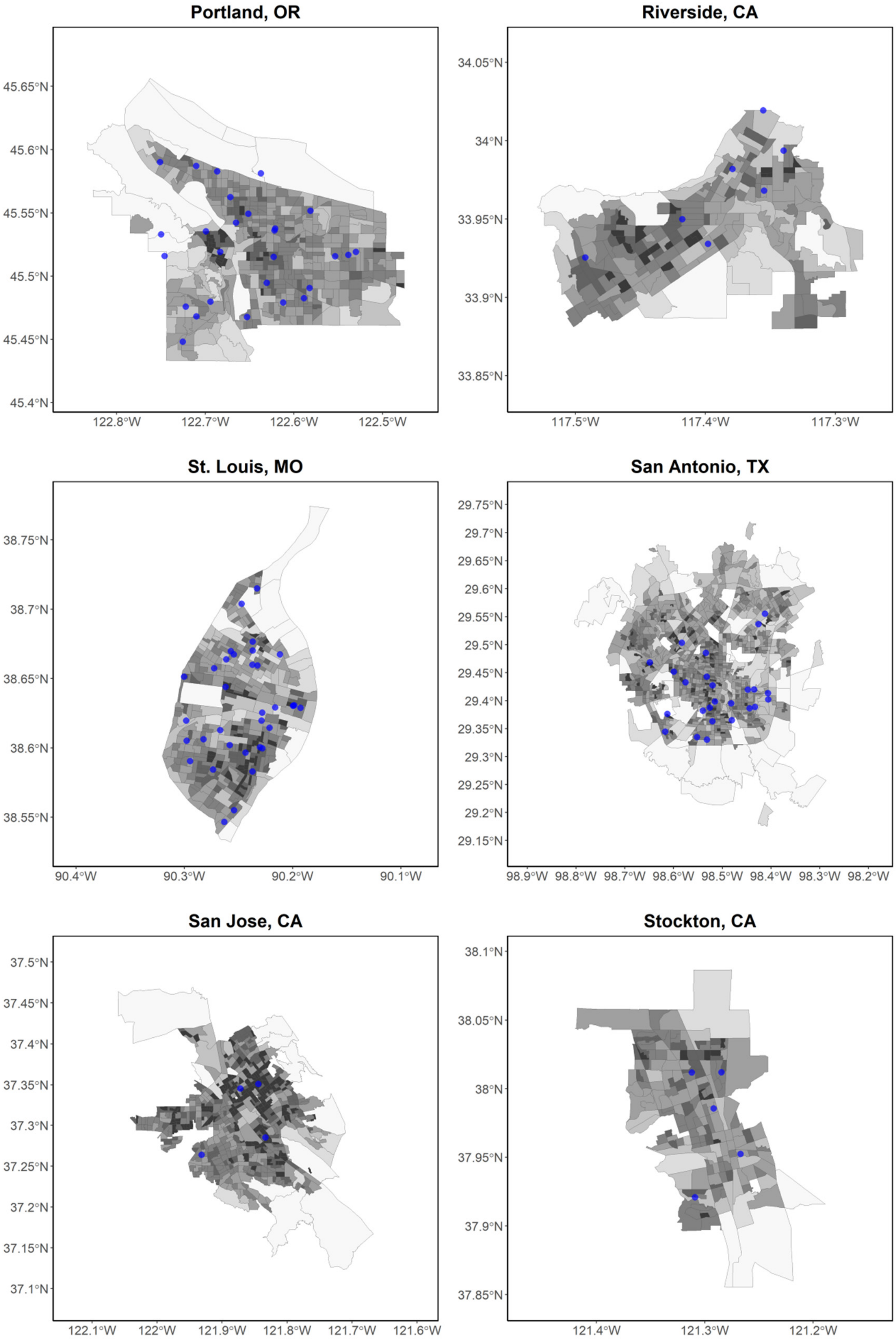
Portland, OR
45.65°N
45.6°N
45.55°N
45.5°N
45.45°N
45.4°N
122.8°W
122.7°W
122.6°W
122.5°W
Riverside, CA
34.05°N
34°N
33.95°N
33.9°N
33.85°N
117.5°W
117.4°W
117.3°W
St. Louis, MO
38.75°N
38.7°N
38.65°N
38.6°N
38.55°N
90.4°W
90.3°W
90.2°W
90.1°W
San Antonio, TX
29.75°N
29.7°N
29.65°N
29.6°N
29.55°N
29.5°N
29.45°N
29.4°N
29.35°N
29.3°N
29.25°N
29.2°N
29.15°N
98.9°W
98.8°W
98.7°W
98.6°W
98.5°W
98.4°W
98.3°W
98.2°W
San Jose, CA
37.5°N
37.45°N
37.4°N
37.35°N
37.3°N
37.25°N
37.2°N
37.15°N
37.1°N
122.1°W
122°W
121.9°W
121.8°W
121.7°W
121.6°W
Stockton, CA
38.1°N
38.05°N
38°N
37.95°N
37.9°N
37.85°N
121.4°W
121.3°W
121.2°W

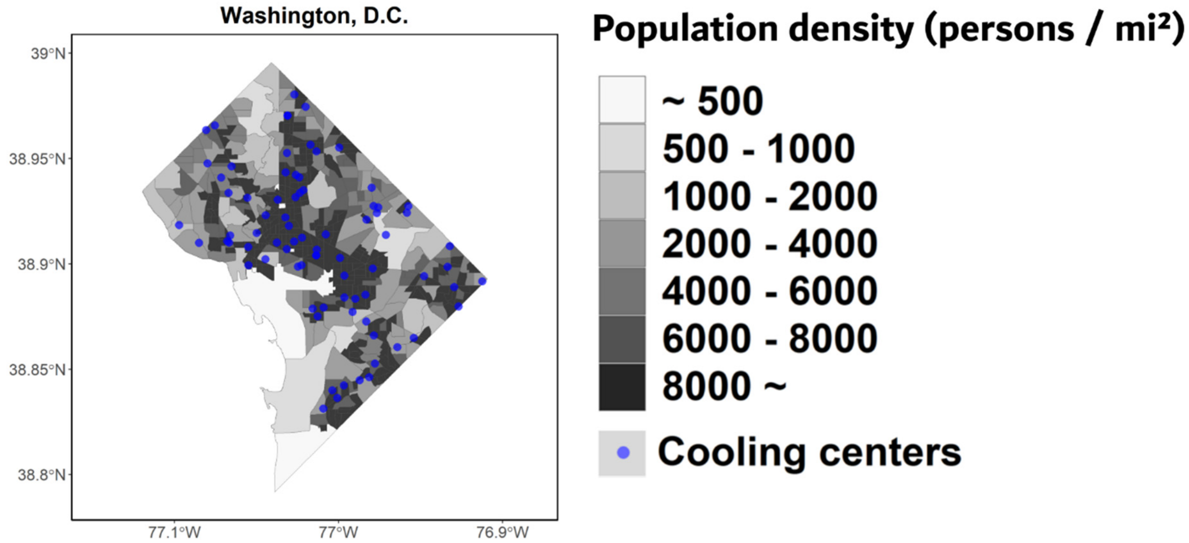

Figure S1. Cooling center locations and population densities.

Table S1. Sources of cooling center in 25 cities.

City	Type	Sources
Albuquerque, NM	City government's websites	https://www.cabq.gov/family/news/visit-one-of-many-public-facilities-to-cool-down-during-albuquerque2019s-heat-wave [Accessed on 10 January 2021]
Baltimore, MD	City government's websites	https://health.baltimorecity.gov/sites/default/files/health/attachments/CodeRedCoolingCenters_5.pdf [Accessed on 25 September 2020]
Chicago, IL	GIS portal	https://data.cityofchicago.org/Health-Human-Services/Cooling-Centers-Map/cj7n-sh49 [Accessed on 10 January 2021]
Columbus, OH	City government's websites	https://www.columbus.gov/recreationandparks/recreation-centers/All-Community-Centers/ [Accessed on 10 January 2021]
Dallas, TX	City government's websites	https://dallascityhall.com/departments/officeemergencymanagement/Pages/Heat-Advisory-Cooling-Stations.aspx [Accessed on 10 January 2021]
Detroit, MI	City government's websites	https://www.waynecounty.com/elected/executive/cooling-centers-in-wayne-county.aspx [Accessed on 25 September 2020]
Fresno, CA	City government's websites	https://www.fresno.gov/parks/cooling-centers/ [Accessed on 10 January 2021]
Kansas City, MO	GIS portal	https://data.mo.gov/Health/Missouri-Cooling-Centers-Map/2wki-9iz8 [Accessed on 25 September 2020]
Long Beach, CA	City government's websites	http://www.longbeach.gov/park/business-operations/about/cooling-center-locations/ [Accessed on 10 January 2021]
Louisville, KY	GIS portal	https://www.arcgis.com/apps/webappviewer/index.html?id=f9b70338e9884006a527fd31655ae85d [Accessed on 10 January 2021]
Memphis, TN	GIS portal	https://tdh.maps.arcgis.com/apps/MapTools/index.html?appid=687ac7d097ea4f67b7be3094ff4e907d [Accessed on 10 January 2021]

Mesa, AZ	GIS portal	https://azmag.maps.arcgis.com/apps/MapSeries/index.html?appid=be7634cb523d4c048106890c98dc3a75&folderid=126f08d6351a4c3a8eb485a11caff1f8 [Accessed on 10 January 2021]
Milwaukee, WI	City government's websites	https://city.milwaukee.gov/health/pageshares/Cooling-Sites.htm [Accessed on 25 September 2020]
Minneapolis, MN	GIS portal	https://www.hennepin.us/residents/health-medical/cooling-centers [Accessed on 10 January 2021]
Nashville, TN	GIS portal	https://tdh.maps.arcgis.com/apps/MapTools/index.html?appid=687ac7d097ea4f67b7be3094ff4e907d [Accessed on 10 January 2021]
Oakland, CA	City government's websites	https://www.shelteroak.org/cooling-centers.html [Accessed on 10 January 2021]
Philadelphia, PA	GIS portal	https://www.arcgis.com/apps/webappviewer/index.html?id=0afe8e198cd84da6a51ca4af027a7056 [Accessed on 10 January 2021]
Phoenix, AZ	GIS portal	https://azmag.maps.arcgis.com/apps/MapSeries/index.html?appid=be7634cb523d4c048106890c98dc3a75&folderid=126f08d6351a4c3a8eb485a11caff1f8 [Accessed on 25 September 2020]
Portland, OR	GIS portal	https://www.arcgis.com/apps/webappviewer/index.html?id=0856c38202954fc19ce55f4d60794324&extent=-13716579.5946,5688527.9073,-13634027.6041,5740734.3976,102100 [Accessed on 25 September 2020]
Riverside, CA	GIS portal	https://www.capriverside.org/Cool-Centers [Accessed on 25 September 2020]
St. Louis, MO	GIS portal	https://www.arcgis.com/apps/webappviewer/index.html?id=a40c7955be2f41f09a4e11520c6ee34b [Accessed on 10 January 2021]
San Antonio, TX	City government's websites	https://www.sanantonio.gov/gpa/News/ArtMID/24373/ArticleID/19170/City-of-San-Antonio-announces-three-cooling-centers-will-open-to-the-public-today-thru-Friday-and-extended-hours-for-some-locations [Accessed on 10 January 2021]
San Jose, CA	City government's websites	https://siliconvalleystrong.org/heat/ [Accessed on 10 January 2021]
Stockton, CA	City government's websites	http://www.stocktonca.gov/government/departments/communityServices/communityCenters.html [Accessed on 10 January 2021]
Washington, DC	GIS portal	https://opendata.dc.gov/datasets/bd51c0c373d844fa9654a7a02da23d6f_0 [Accessed on 10 January 2021]

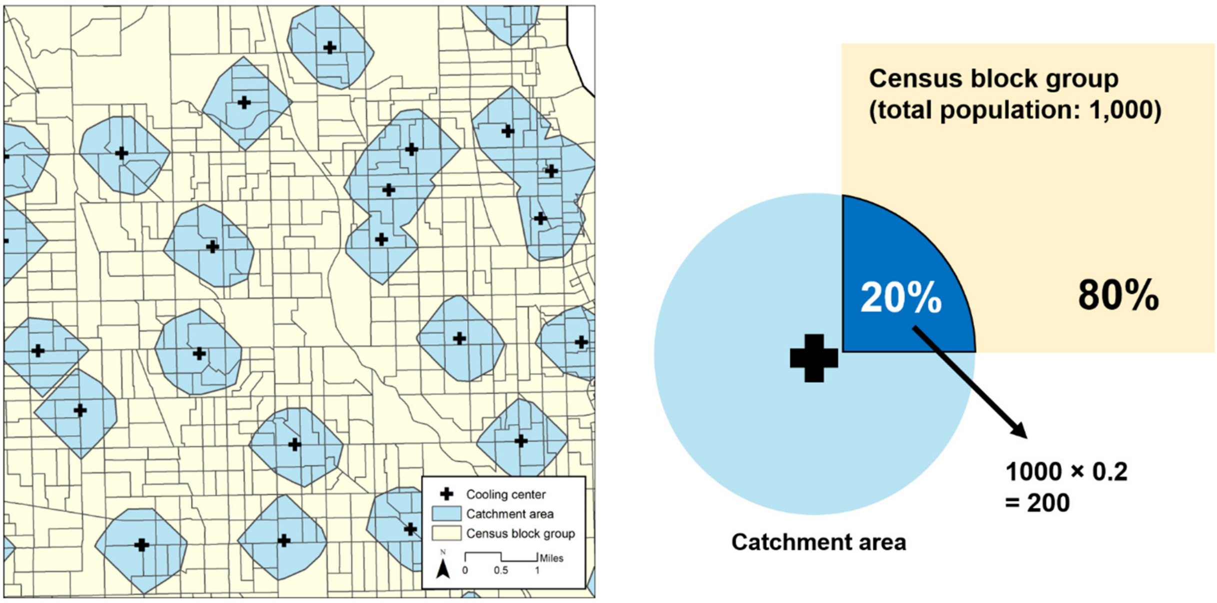

Figure S2. Illustration of catchment areas.

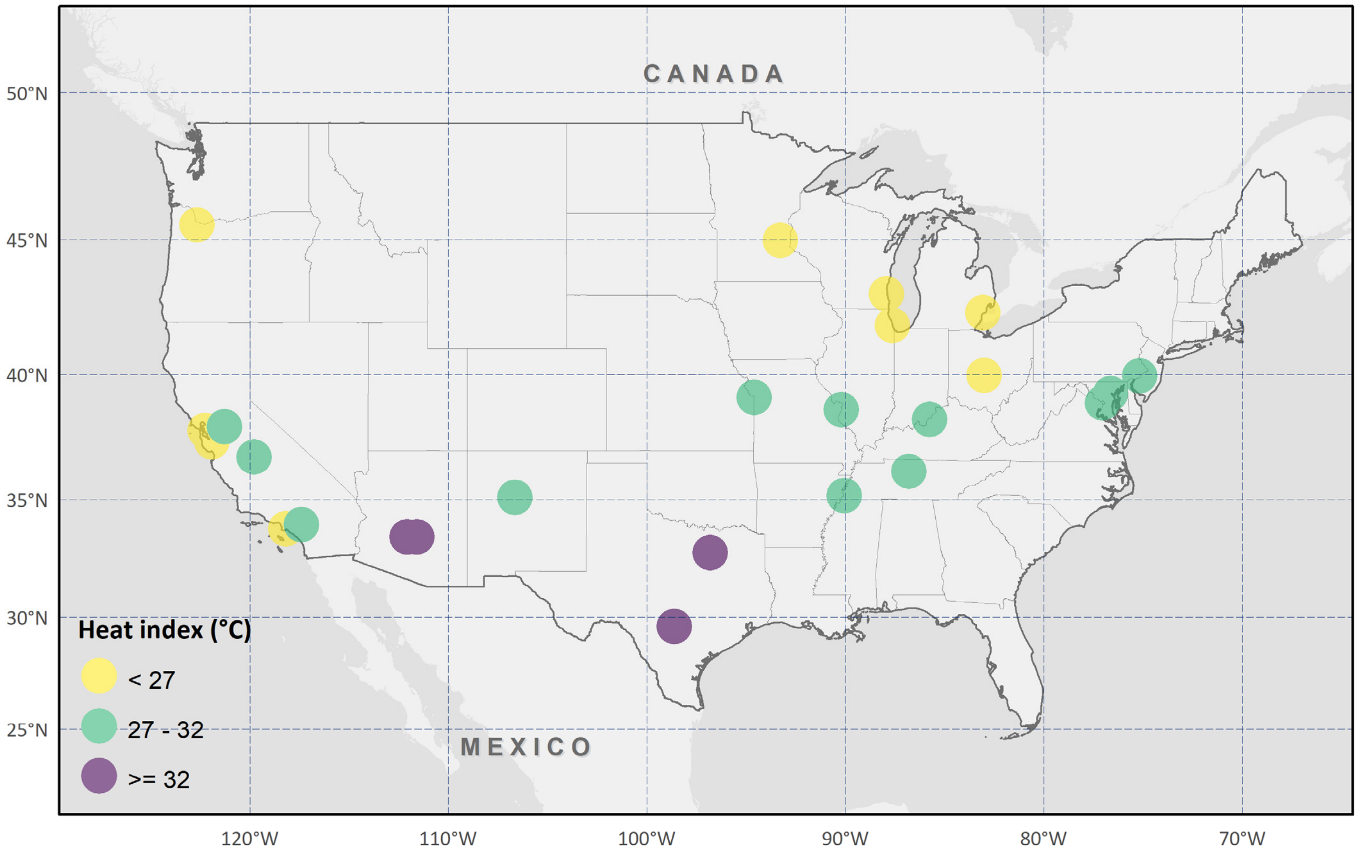

Figure S3. Spatial distribution of heat index.

Table S2. Total population coverage and standardized population coverage of study cities.

City	Total Population Coverage (%)	Standardized Population Coverage (%)
Albuquerque, NM	13.3	0.3
Baltimore, MD	10.4	0.9
Chicago, IL	25.9	0.3
Columbus, OH	6.7	0.2
Dallas, TX	6.7	0.1
Detroit, MI	9.4	0.4
Fresno, CA	1.9	0.5
Kansas City, MO	8.1	0.3
Long Beach, CA	7.0	1.4
Louisville, KY	5.2	0.2
Memphis, TN	3.3	0.2
Mesa, AZ	2.1	0.3
Milwaukee, WI	9.8	0.6
Minneapolis, MN	5.7	1.0
Nashville, TN	2.7	0.2
Oakland, CA	7.0	1.4
Philadelphia, PA	17.6	0.6
Phoenix, AZ	3.0	0.1
Portland, OR	13.0	0.5
Riverside, CA	4.4	0.6
St. Louis, MO	27.1	0.7
San Antonio, TX	3.0	0.1
San Jose, CA	2.1	0.4
Stockton, CA	3.9	0.8
Washington, DC	57.5	0.7
Average	10.3	0.5
Standard deviation	11.9	0.4

Table S3. Separate SPCs for selected subpopulation groups and reference groups.

City	Age		Black		Hispanic		Insurance		Poverty	
	≥65	≤64 (Ref)	Yes	No (Ref)	Yes	No (Ref)	No	Own (Ref)	Below	Above (Ref)
Albuquerque, NM	0.30	0.29	0.36	0.29	0.31	0.28	0.33	0.29	0.41	0.27
Baltimore, MD	0.91	0.95	0.84	1.11	1.04	0.94	1.08	0.94	1.15	0.91
Chicago, IL	0.25	0.26	0.25	0.27	0.26	0.26	0.27	0.26	0.27	0.26
Columbus, OH	0.21	0.23	0.28	0.21	0.26	0.23	0.27	0.23	0.35	0.20
Dallas, TX	0.16	0.15	0.12	0.16	0.16	0.15	0.15	0.15	0.13	0.16
Detroit, MI	0.40	0.39	0.34	0.60	0.35	0.40	0.40	0.39	0.36	0.39
Fresno, CA	0.42	0.47	0.61	0.46	0.66	0.29	0.66	0.46	0.80	0.37
Kansas City, MO	0.26	0.30	0.34	0.27	0.28	0.29	0.33	0.28	0.34	0.28
Long Beach, CA	1.06	1.45	1.57	1.37	1.68	1.19	1.83	1.36	2.12	1.27
Louisville, KY	0.19	0.21	0.43	0.14	0.13	0.21	0.20	0.21	0.44	0.17
Memphis, TN	0.19	0.18	0.20	0.14	0.19	0.18	0.19	0.18	0.24	0.16
Mesa, AZ	0.16	0.32	0.30	0.30	0.54	0.20	0.43	0.28	0.49	0.26
Milwaukee, WI	0.58	0.62	0.76	0.52	0.30	0.69	0.48	0.61	0.66	0.55
Minneapolis, MN	0.86	0.95	1.96	0.72	1.31	0.92	1.53	0.89	1.65	0.74
Nashville, TN	0.14	0.21	0.42	0.13	0.09	0.22	0.14	0.22	0.52	0.15
Oakland, CA	1.43	1.41	1.29	1.44	1.45	1.38	1.85	1.36	1.86	1.31
Philadelphia, PA	0.56	0.62	0.57	0.63	0.60	0.61	0.62	0.61	0.66	0.59
Phoenix, AZ	0.08	0.10	0.14	0.10	0.12	0.09	0.13	0.09	0.15	0.08
Portland, OR	0.45	0.49	0.49	0.48	0.42	0.49	0.50	0.48	0.51	0.48
Riverside, CA	0.58	0.62	0.74	0.61	0.73	0.49	0.81	0.60	0.82	0.61
St. Louis, MO	0.70	0.74	0.75	0.72	0.69	0.73	0.70	0.73	0.73	0.73
San Antonio, TX	0.13	0.12	0.13	0.12	0.15	0.06	0.16	0.11	0.18	0.11
San Jose, CA	0.32	0.42	0.24	0.42	0.82	0.23	0.68	0.40	0.52	0.41
Stockton, CA	0.87	0.79	0.76	0.79	0.78	0.79	0.77	0.78	0.97	0.73
Washington, DC	0.68	0.74	0.63	0.81	0.80	0.72	0.72	0.73	0.66	0.75
Average	0.48	0.52	0.58	0.51	0.56	0.48	0.61	0.51	0.68	0.48
